# Absence of the Caspases 1/11 Modulates Liver Global Lipid Profile and Gut Microbiota in High-Fat-Diet-Induced Obese Mice

**DOI:** 10.3389/fimmu.2019.02926

**Published:** 2020-01-09

**Authors:** Lívia Pimentel de Sant'Ana, Dalila Juliana S. Ribeiro, Aline Maria Araújo Martins, Fábio Neves dos Santos, Rafael Corrêa, Raquel das Neves Almeida, Marcos Nogueira Eberlin, Corinne F. Maurice, Kelly Grace Magalhães

**Affiliations:** ^1^Laboratory of Immunology and Inflammation, Department of Cell Biology, University of Brasilia, Brasília, Brazil; ^2^CEMBIO—Centro de Metabolómica y Bioanálisis, Universidad San Pablo CEU, Madrid, Spain; ^3^ThoMSon Mass Spectrometry Laboratory, Institute of Chemistry, University of Campinas—UNICAMP, São Paulo, Brazil; ^4^Department of Chemistry, Mackenzie University, São Paulo, Brazil; ^5^Department of Microbiology and Immunology, McGill University, Montreal, QC, Canada

**Keywords:** obesity, gut microbiota, high-fat diet, NAFLD, inflammasome

## Abstract

Obesity is a chronic disease with rising worldwide prevalence and largely associated with several other comorbidities, such as cancer, non-alcoholic fatty liver disease (NAFLD), and metabolic syndrome. Hepatic steatosis, a hallmark of NAFLD, is strongly correlated with obesity and has been correlated with changes in the gut microbiota, which can promote its development through the production of short-chain fatty acids (SCFAs) that regulate insulin resistance, bile acid, choline metabolism, and inflammation. Recent studies have suggested a controversial role for the inflammasome/caspase-1 in the development of obesity and non-alcoholic steatohepatitis (NASH). Here, we evaluated the role of inflammasome NLRP3 and caspases 1/11 in the establishment of obesity and hepatic steatosis in diet-induced obese mice, correlating them with the global lipid profile of the liver and gut microbiota diversity. After feeding wild-type, caspases 1/11, and NLRP3 knockout mice with a standard fat diet (SFD) or a high-fat diet (HFD), we found that the caspases 1/11 knockout mice, but not NLRP3 knockout mice, were more susceptible to HFD-induced obesity, and developed enhanced hepatic steatosis even under SFD conditions. Lipidomics analysis of the liver, assessed by MALDI-MS analysis, revealed that the HFD triggered a significant change in global lipid profile in the liver of WT mice compared to those fed an SFD, and this profile was modified by the lack of caspases 1/11 and NLRP3. The absence of caspases 1/11 was also correlated with an increased presence of triacylglycerol in the liver. Gut microbial diversity analysis, using 16S rRNA gene sequencing, showed that there was also an increase of Proteobacteria and a higher Firmicutes/Bacteroidetes ratio in the gut of caspases 1/11 knockout mice fed an HFD. Overall, mice without caspases 1/11 harbored gut bacterial phyla involved with weight gain, obesity, and hepatic steatosis. Taken together, our data suggest an important role for caspases 1/11 in the lipid composition of the liver and in the modulation of the gut microbial community composition. Our results further suggest that HFD-induced obesity and the absence of caspases 1/11 may regulate both lipid metabolism and gut microbial diversity, and therefore may be associated with NAFLD and obesity.

## Introduction

Obesity has reached epidemic proportions globally according to the World Health Organization, since the number of obese individuals has been dramatically increasing worldwide. Obesity is defined as abnormal or excessive fat accumulation that presents a significant danger to health, increasing the risk of type 2 diabetes, hypertension, and hyperlipidemia, among others. This metabolic disorder results from an imbalance in energy intake, energy expense, and fat aggregation (1). Progression of obesity is followed by the development of a chronic inflammation promoted by the white adipose tissue and the influx of macrophages and T cells in this tissue.

Among the several factors related to the progression and pathogenesis of obesity, many studies have speculated about the role of gut microbiota and non-alcoholic fatty liver disease (NAFLD). The gut microbiome is central to the maturation and development of mucosal and systemic immunity, the protection of the host against pathogens, and the conservation of intestinal epithelial homeostasis (2). The gut microbiota is at the interface of host nutrition, genetics, and energy balance. Alterations in gut microbial diversity, also termed dysbiosis, have been associated with the development of obesity, and associated metabolic conditions.

Intestinal dysbiosis is characterized by significant shifts in microbial composition, abundance, diversity, metabolism, and function (3). This state can result from a variety of conditions: intestinal infection, altered host genetics, inflammation, and dietary changes (4, 5). In obesity, the overall composition of the gut microbiota has been reported as altered in several studies in animal models and human subjects, with an increase in the *Firmicutes/Bacteroidetes* (F/B) ratio in genetically obese mice (*ob/ob*) (6) and in mice fed a high-fat diet (HFD) (7). Moreover, germ-free (GF) mice presented reduced levels of total body fat relative to conventionally raised (CONV-R) mice. Additionally, the transfer of the gut microbiota from CONV-R to GF mice increased the body fat content by 60% and insulin resistance of the host (8). Comparisons of the metabolite profiles of mice fed a HFD and low-fat diet (LFD) identified some microbial-derived metabolites that are depleted after HFD (9).

The characteristic inflammatory status in obesity triggers the release of pro-inflammatory cytokines, chemokines, and pro-inflammatory fatty acids (10). Two of the pro-inflammatory cytokines already linked to obesity are IL-1β and IL-18 (11). These cytokines are a direct product of inflammasome activation by the cleavage of caspase 1. Inflammasomes are multi-protein platforms containing one of many upstream NOD-like receptor (NLR) proteins, which may or may not contain the adaptor protein apoptosis-associated speck-like protein containing CARD (ASC), and the effector caspase 1 (12). Caspase 1 knockout mice kept under HFD has been shown to develop obesity due to reduced IL-18 levels (13). Murine and human models revealed that increased NLRP3 expression in adipose tissue is linked to obesity-associated insulin resistance (14). Thus, in the context of obesity and NAFLD, inflammasomes not only orchestrate host defense mechanisms during infection, but their contribution to innate responses also includes the control of the gut microbiota composition (15).

A complex balance exists among diet, gut microbiota diversity, obesity, and the regulation of immune and inflammatory responses. There is a growing concern that diet-induced changes in the gut microbiota can directly contribute to the growing epidemics of metabolic diseases. However, the mechanisms through which gut bacteria respond to dietary changes and how other inflammatory complexes alter this response remain unclear. Thus, in this work, we aimed to elucidate how the absence of caspases 1/11 can influence the effects of HFD-induced obesity in mouse models by exploring changes in gut microbial composition and liver global lipid profile.

## Materials and Methods

### Animal Work

Wild-type (WT) 8-week-old female mice C57BL/6J were used, as well as knockouts for caspase 1/11 (*Caspases 1/11*^−/−^) and NLRP3 (*Nlrp3*^−/−^). *Caspases 1/11*^−/−^ and *Nlrp3*^−/−^ mice were kindly provided by Prof. Dario S. Zamboni from the University of São Paulo–Ribeirão Preto. Mice were kept in the Laboratory Animal Breeding and Experimental Facility of the Institute of Biological Sciences of the University of Brasília throughout the experiments and the absence of NLRP3 and caspase 1 and 11 was periodically checked by genotyping. Animals were housed under 12-h light–dark cycles at a controlled temperature (23°C ± 2°C), with water and food *ad libitum*. Sixteen animals from each genotype (WT, *Caspases1/11*^−/−^, or *Nlrp3*^−/−^) were randomized into 2 groups (*n* = 8 per group) and treated for 90 days with standard fat diet (SFD) or high fat diet (HFD). The SFD was AIN-93G (16) (17 kcal% fat – 83% unsaturated fat and 16% saturated fat). The HFD was AIN-93G modified (45 kcal% fat – 59% unsaturated fat and 41% saturated fat).

### Histological Analysis

Livers were fixed in 3.7% formaldehyde overnight at room temperature immediately after euthanasia. Organs were paraffinized and sectioned at 5-μm thickness by a microtome. The slides were stained with hematoxylin and eosin (HE) (Sigma) following standard procedures (17). Sections were examined by light microscopy Zeiss Lab. A1 Axiocam 105 color and photomicrographs were scanned using the ZEN program from Zeiss. Steatosis was numerically scored following semi-quantitative pathological standards using ImageJ software (18).

### Mass Spectrometry Analysis

#### Lipids Extraction Procedure From Livers

We used a modification of the Bligh-Dyer protocol for lipid extraction method (19). Briefly, the liquid was suspended in 150 μl of Milli-Q water. Then, 190 μl of chloroform and 375 μl of methanol were added and the mixture was vortexed for 5 min. Then, 190 μl of chloroform and 150 μl of Milli-Q water were added and vortexed for 1 min, and the mixture was centrifuged at 14,000 × *g* for 5 min to induce phase separation. The lower organic layer was collected, concentrated in a Speed-Vac apparatus, and reconstituted into 100 μl of a chloroform/methanol (1:1) solution. The mixture was then spotted (1 μl droplet) onto a MALDI plate and air-dried. Then, 1 μl of 2,5-dihydroxybenzoic acid (DHB) matrix, prepared at a concentration of 10 mg ml^−1^ in methanol, was spotted over the dried sample. The TOF analyzer calibration and tuning were performed using a phospholipid mixture composed of DOPE, DMPG, DPPA, and DPPS for lipid analysis.

#### MALDI-MS Analysis

MALDI-MS was performed on a Bruker Autoflex III MALDI–TOF/TOF mass spectrometer equipped with a 334-nm smart beam laser. The spectrum was acquired in the TOF linear mode and in the positive ionization mode with a delayed extraction of 260 ns at 20 kV accelerating voltage. Each spectrum was manually collected as an average of 5,000 laser shots (1,000 laser shots at five different spot positions). Laser energy was set just at 70%. A range of *m/z* 600–1,200 was used to obtain the lipid profiles. Spectra were acquired in triplicate and the AutoExecute tool of Flexcontrol acquisition software was used (Version 2.4; Bruker-Daltonik GmbH) for processing. Only ions with an S/N ratio >3 were considered.

#### Multivariate Analysis of MALDI-MS Data

The analysis of MALDI data was done via three distinct steps: (1) pre-processing, (2) processing, and (3) statistical analysis. Raw spectra were pre-processed in the FlexAnalysis software (Bruker-Daltonik) for matrix background removal, alignment of the spectra scale, ion selection with an S/N ratio >3, and normalization of abundances. Data processing was performed before multivariate analysis for the lipid profiles in the MetaboAnalyst 3.0 (version software). The uploaded files (.csv format) comprised a list of ions (*m/z* and relative abundances). The ions were realigned within a tolerance of *m/z* 0.4 (0.4 Da) to remove ions that appear in less than half of the samples in each group. The presence of missing values or ions with constant values (i.e., all zeros) was checked and data filtering using relative standard deviation (RSD) was applied to remove variables close to baseline or detection limit as variables that are near-constant values. Then, the relative abundance of ion was normalized by autoscaling (mean-centered and divided by the standard deviation of each variable) as preprocessing to multivariate analysis. Both unsupervised and supervised statistical approaches were applied. Principal component analysis (PCA) and partial least squares–discriminant analysis (PLS-DA) were performed on the data using the MetaboAnalyst (version 3.0 software) (20) to discriminate the samples based on their lipid profiles. To determine independent factors and relevant ions within the experiment, we used Variable Importance in Projection (VIP) from PSL-DA analysis. All data were obtained from a non-parametric analysis.

#### Annotation of Lipids

The major discriminant lipids of the MALDI-MS analysis were selected out of the multivariate analysis and their annotation was made according to the exact mass (*m/z*) of the protonated ion and their fragmentation spectra. The lipid data (.txt format) comprised a *m/z* and relative abundance list of the ions obtained from MS and MS/MS spectra were uploaded to Metlin (http://metlin.scripps.edu), ChemSpider (www.chemspider.com), and MassBank (http://www.massbank.jp/); The Human Metabolome DataBase—HMDB (21) and the LIPID Metabolites And Pathways Strategy—LIPID MAPS® (Wellcome Trust) spectra database were used to annotate the lipids.

### Gut Microbial Analysis

#### DNA Extraction and Amplification of V4 Region

Mice fecal samples from all groups were collected at the end of treatment and immediately stored at −80°C. High-throughput sequencing of the V4 region of the 16S ribosomal RNA (rRNA) gene was performed to characterize the distal gut microbiota composition, according to published works (22). Each sample was subjected to DNA extraction with MoBio PowerSoil DNA isolation kit protocol (MoBio, Carlsbad, CA, USA), before quantification with a Nanodrop. The V4 region of the 16S rRNA gene was PCR-amplified in triplicate with custom barcoded universal bacterial primers using the following protocol: 94°C for 3 min, 35 cycles of 94°C for 45 s, 50°C for 30 s, and 72°C for 90 s, with a final extension at 72°C for 10 min (4), and sequenced on an Illumina HiSeq platform at the McGill University and Genome Quebec Innovation Center. The quality of the run was analyzed on a 1.5% agarose gel, and controls consisted of the PCR reaction without the DNA template. 16S rRNA gene sequences were analyzed using the QIIME software package (23).

### Statistical Analysis

Results are reported as mean ± SD, or mean ± SEM unless otherwise noted. Differences among the groups were compared using ANOVA with Turkey post-test for multiple comparisons and Student's *t* test with Bonferroni corrections when there were only two groups. All statistical analysis was performed using GraphPad PRISM 6 or QIIME software. *p*-values are represented by asterisks: *p* ≤ 0.05 (^*^), *p* ≤ 0.01 (^**^), *p* ≤ 0.001 (^***^), and *p* ≤ 0.0001 (^****^).

### Ethics Approval Statement

This study was approved by the Committee for Ethics in Animal Use of the Institute of Biological Sciences of the University of Brasilia, UnBDoc n°52306/2014.

## Results

### The Absence of Inflammasome Components Directly Influences Weight Gain Under a HFD

**Figure 1:**
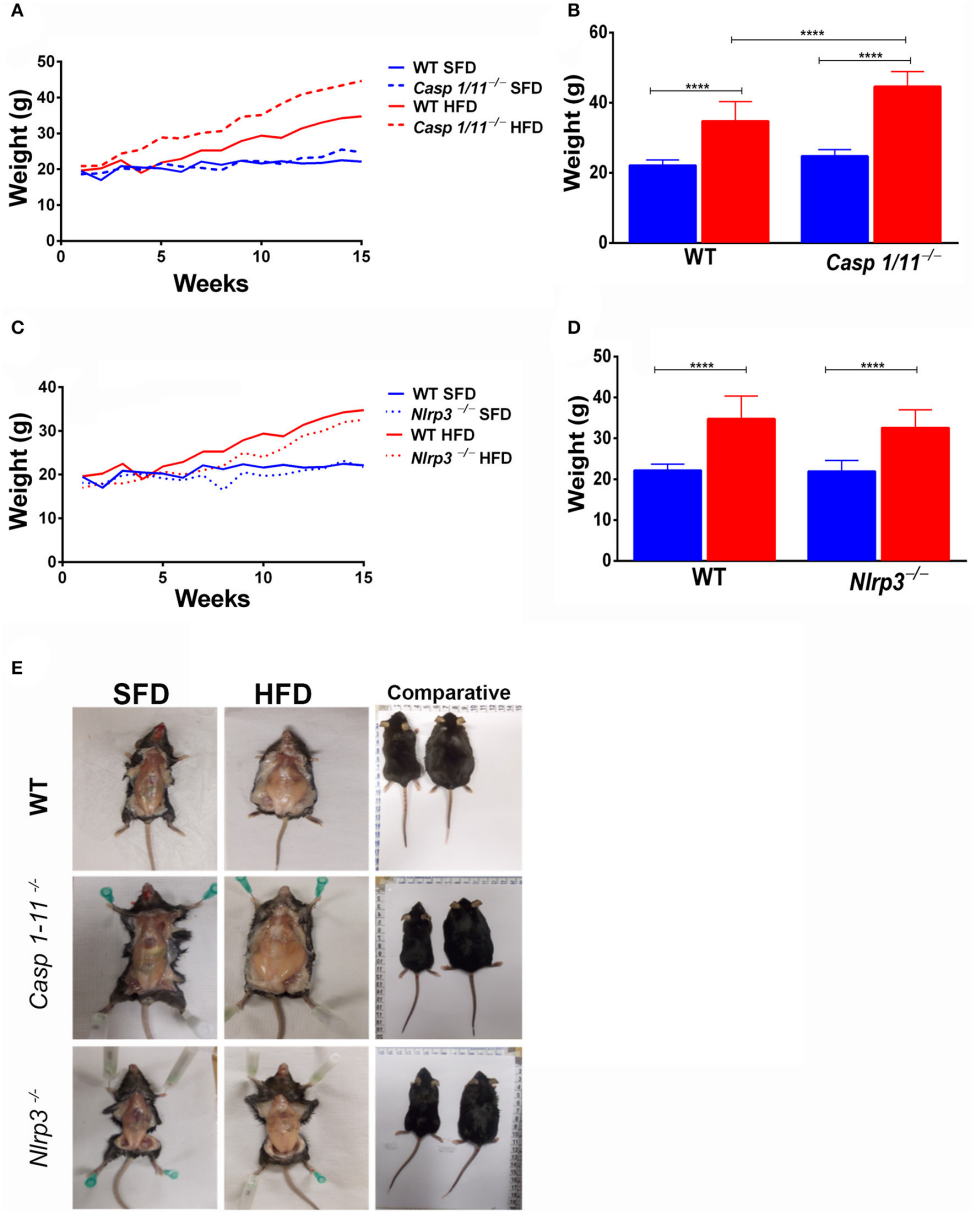
Establishment of high-fat-diet-induced obesity. WT, *caspase-1/11*^−/−^ and *Nlpr3*^−/−^ mice were fed SFD (17% fat) or HFD (45% fat) for 90 days. The weight gain curve was fitted with the weekly weighing of the animals **(A,C)**. Weight of the animals at the final of treatment **(B,D)** and abdominal fat **(E)**. ANOVA test was applied, the bars representing confidence interval of 95% (****p < 0.0001).

To investigate the influence of inflammasome components in inducing obesity in response to HFD, C57/BL6 WT, *Caspases 1/11*^−/−^, and *Nlrp3*^−/−^ mice were submitted to SFD or HFD for 3 months, with weekly monitoring of weight gain (Figure 1A). All animals fed the HFD showed an increase in weight (Figures 1C,D). This increase was faster and more pronounced in mice lacking caspases 1 and 11, with a significant increase in the final weight of *Caspases 1/11*^−/−^ mice compared to the WT mice (Figure 1C). The absence of NLRP3 protein did not induce weight gain compared to WT mice, as WT mice presented a greater weight than *Nlrp3*^−/−^, despite not being significant (Figure 1D). WT, *Caspases 1/11*^−/−^, and *Nlrp3*^−/−^ mice presented no difference in SFD or HFD food intake (Supplementary Figure S1).

### Lack of Caspases 1/11 Alters Steatosis Levels in the Liver After HFD

**Figure 2:**
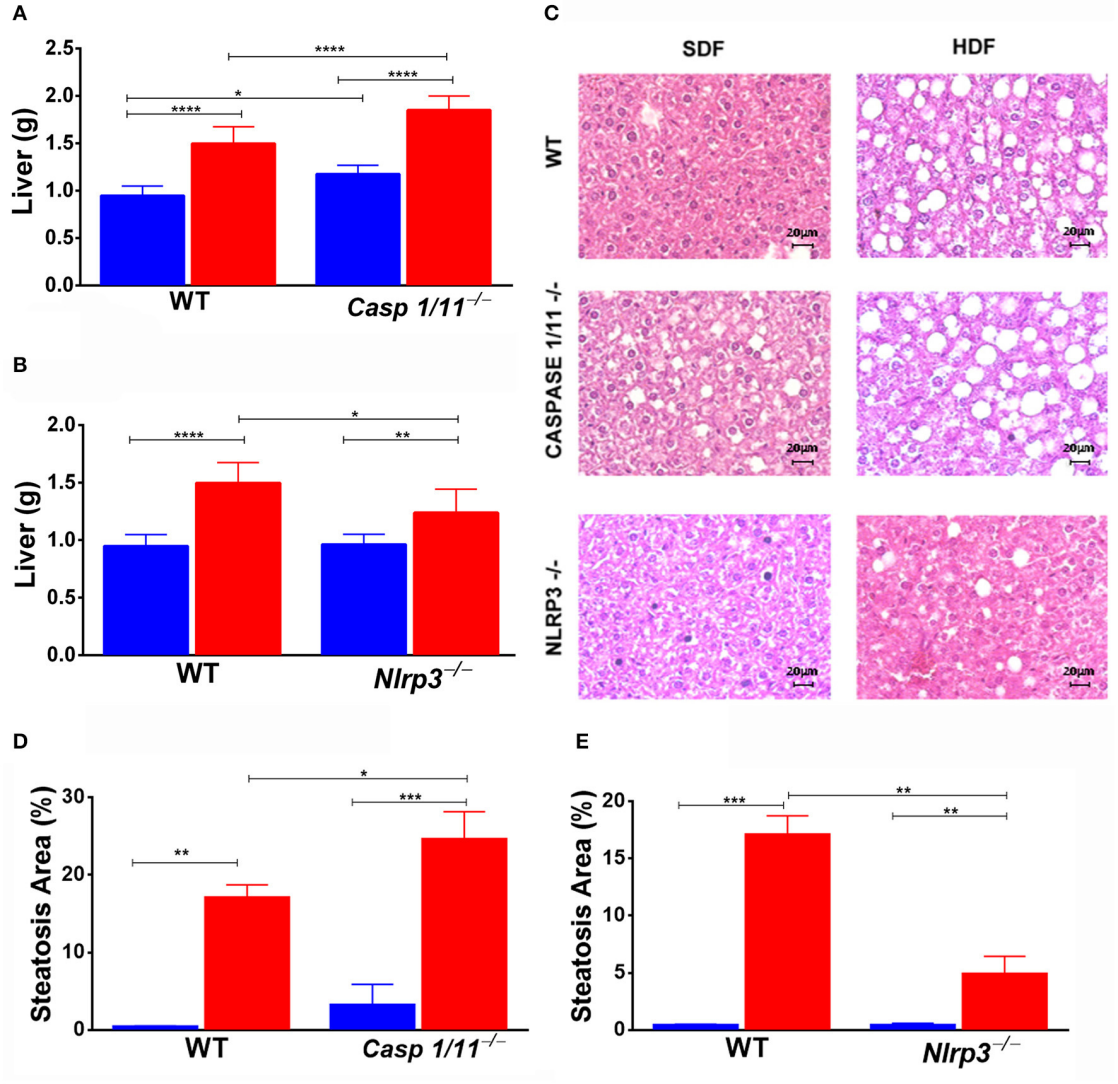
Weight of organs and hepatic steatosis development. WT, *caspase-1/1*^−/−^ and *Nlpr3*^−/−^ mice were fed SFD (17% fat) or HFD (45% fat) for 90 days; after treatment, the liver was collected. Weight of liver **(A,B)** and hematoxylin and eosin staining of the liver **(C)** after treatment. Hepatic steatosis scores **(D,E)** were determined with semi-quantitative pathological standards. ANOVA test was applied, the bars representing confidence interval of 95% (*p < 0.05, **p < 0.01, ***p < 0.005, and ****p < 0.0001).

In the animals fed a HFD, we observed a significant increase in the liver weight of *Caspases 1/11*^−/−^ mice compared to both WT (Figure 2A) and *Nlrp3*^−/−^ mice (Figure 2B). *Caspases 1/11*^−/−^ mice fed a SFD already presented a fat accumulation in the liver, in addition to the increased weight of the liver compared to WT mice fed a SFD (Figure 2A). Non-alcoholic fatty liver disease (NAFLD), which strongly correlates with obesity and metabolic syndrome, is primarily characterized by hepatic steatosis (24). To assess whether the increased liver weight observed could be related to fat accumulation, we evaluated the hepatic fat content through HE staining. This histologic imaging showed that the HFD induced fat accumulation, with features of micro- and macro-vesicular steatosis in all genotypes. *Caspases 1/11*^−/−^ mice presented a more significant presence of liver steatosis compared to both WT and *Nlrp3*^−/−^ mice (Figures 2C–E).

### HFD Changes the Pattern of Liver Global Lipid Composition in the Absence of NLRP3 and Caspases 1/11

**Figure 3:**
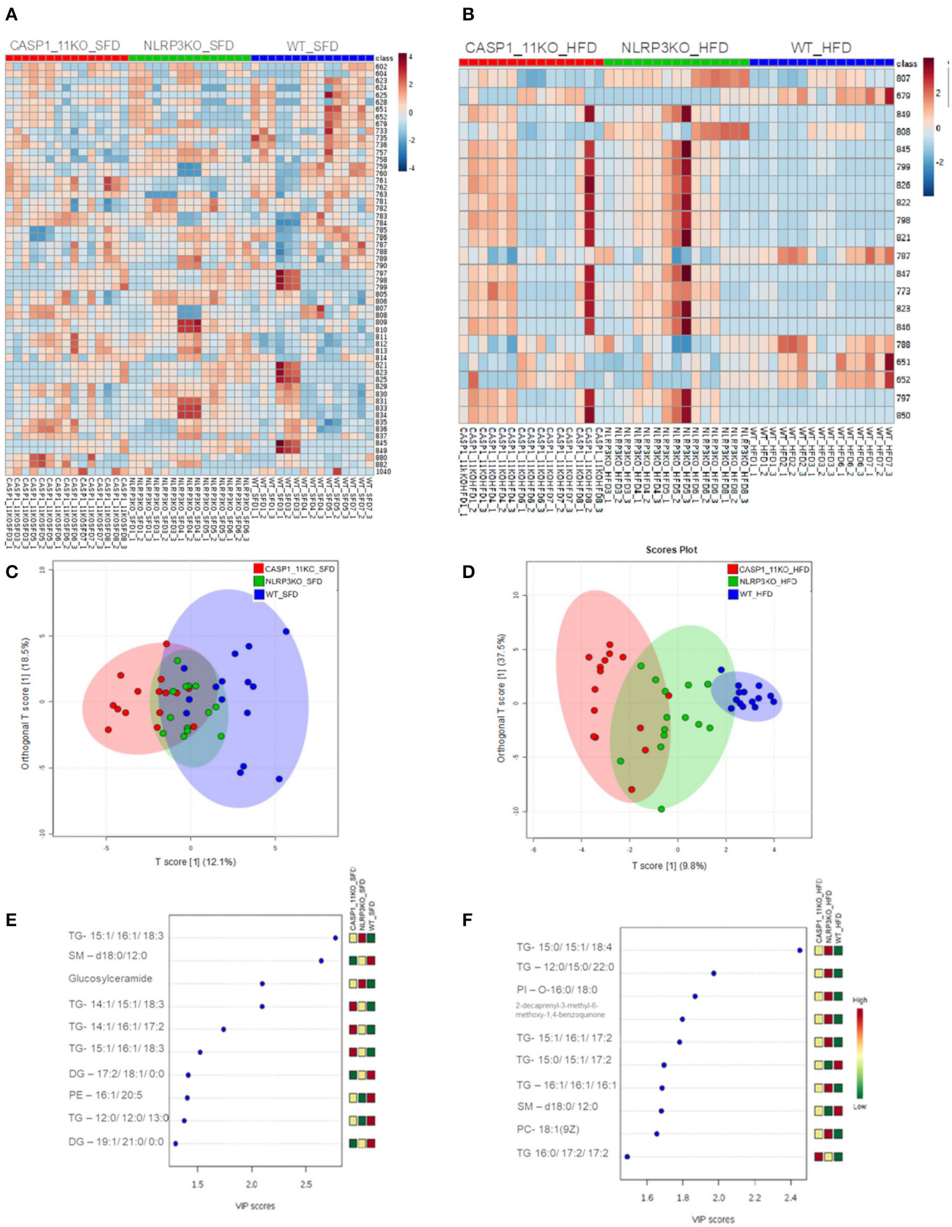
MS/MS analysis of lipid composition in the liver. Heat map, with a color-code thermometer indicating the relative concentrations of metabolites on each group: WT, *caspase-1/11*^−/−^, and *Nlpr3*^−/−^ mice fed SFD (17% fat) or HFD (45% fat) for 90 days. Maps show the lipidomic profiles of SFD **(A)** and HFD **(B)** groups. Orthogonal partial least squares–discriminant analysis (OPLS-DA) score plots model was constructed using MALDI lipid profiles. Each of the five animals in each group gave rise to three samples. Plots show the lipidomic profiles animals fed with SFD **(C)** or HFD **(D)**. Variable importance in projection (VIP) plot identified by partial least squares–discriminant analysis (PLS-DA) displaying the top 10 most important features in mice fed with SFD **(E)** or HFD **(F)**.

To investigate the mechanisms behind the increased hepatic steatosis in *Caspases 1/11*^−/−^ mice, we investigated the liver global lipid profile after HFD using mass spectrometry. In the heat map obtained from the hierarchical cluster analysis (HCA) of SFD groups (Figure 3A), we observed that the three mouse genotypes already demonstrated a different pattern in lipid composition, even in mice under SFD. In the WT mice under SFD, we observed a higher abundance of ions in range *m/z* 600–800, especially when compared with the *Caspases 1/11*^−/−^ mice under SFD. In the orthogonal projections to latent structures–discriminant analysis (OPLS-DA) score plot (Figure 3C), we observed that lipids from WT and *Caspases 1/11*^−/−^ mice tend to form two clusters under SFD, despite the first and second components explaining less than of total variance. Comparing WT and *Nlrp3*^−/−^ mice, while there is a difference in the abundance of some ions (Figures 3A,E), the general view from the OPLS-DA showed an almost total overlap of the two groups. A VIP score of >1 from the PLS-DA model was used as a criterion to determine the discriminating lipids associated with the differentiation of the three genotypes. The most relevant lipids in the VIP score, with a score >2, from the SFD (Figure 3E) showed a majority of triacylglycerols, followed by sphingolipids and glucosylceramides, already from HFD (Figure 3F) showed only of triacylglycerols for the same score.

Mice fed a HFD showed a complete change of liver global lipid pattern, with some of the lipid content being much more abundant in the *Caspases 1/11*^−/−^ mice compared to WT, as observed in the heat map plot (Figure 3B). Only the lipids with a *m/z* of 651.53, 652, and 788 were more abundant in the WT. WT, *Caspases 1/11*^−/−^, and *Nlrp3*^−/−^ mice fed a SFD were apparently overlapped on the PLS-DA score plot (Figure 3C). However, HFD clearly influenced the liver global lipid profile, since we observed that WT, *Caspases 1/11*^−/−^, and *Nlrp3*^−/−^ mice fed a HFD were well-clustered on the PLS-DA score plot (Figure 3D). This difference was even more pronounced when we compare WT and *Caspases 1/11*^−/−^ given the clear separation of two clusters due to the highest data variance in PLS-DA (47.3%), suggesting that absence of caspase 1 and 11 may significantly impact the liver global lipid composition of mice specially fed a HFD. The VIP score revealed that triacylglycerols were the most relevant class of lipids driving these differences.

### HFD Changes the Gut Microbiota Composition in Different Ways in WT and *Caspases 1/11^−/−^* Mice

Deficiency in the NLRP6 inflammasome pathway promotes the progression of an altered and colitogenic gut microbial community (25). Since intestinal microbial dysbiosis is another factor in NAFLD pathogenesis and the liver–gut axis has a strong link with obesity, we also investigated alterations in the gut microbiota. We quantified microbial diversity within each group (α-diversity), and identified that *Caspases 1-11*^−/−^ mice fed a HFD presented a higher gut bacterial diversity (Supplementary Figure 2A). This unexpected result suggested a functional role of caspases 1 and 11 regulating gut bacterial diversity in HFD-induced obese mice. *Caspases 1/11*^−/−^ mice fed HFD harbored once again a more diversified microbial composition compared to WT under the same diet (Supplementary Figure 2B).

**Figure 4:**
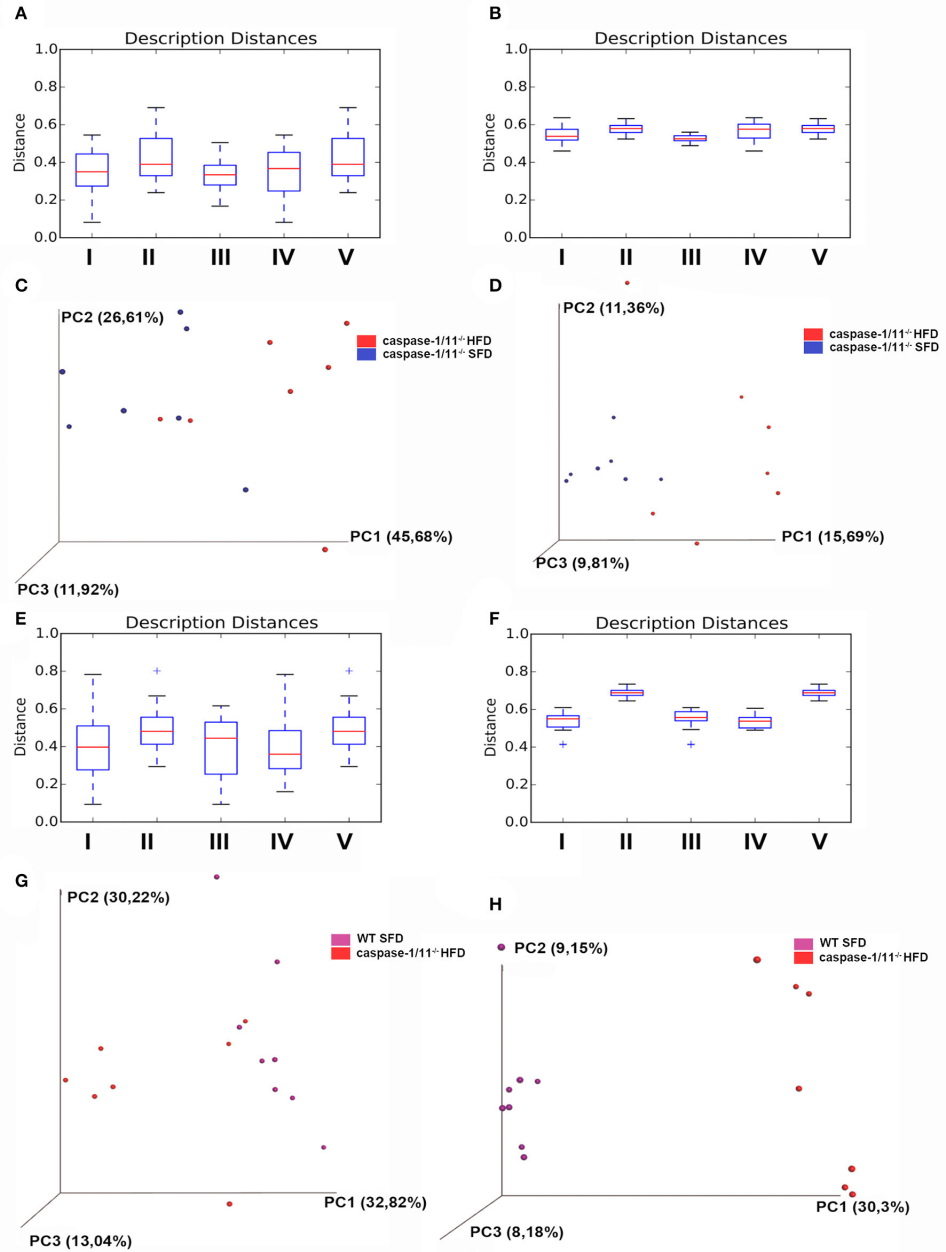
β-diversity of gut microbiota. Distance boxplots weighted_unifrac **(A)** and unweighted unifrac **(B)**: I—All within the description, II—All between description, III—*caspase-1/11*^−/−^ SFD vs. *caspase-1/11*^−/−^ SFD, IV—*caspase-1/11*^−/−^ HFD vs. *caspase-1/11*^−/−^ HFD, and V—*caspase-1/11*^−/−^ SFD vs. *caspase-1/11*^−/−^ HFD. **(C)** PCoA plot weighted_unifrac and **(D)** PCoA plot unweighted_unifrac, blue: *caspase-1/11*^−/−^SFD, red: *caspase-1/11*^−/−^ HFD. Distance boxplots weighted_unifrac **(E)** and unweighted unifrac **(F)**: I—All within the description, II—All between description, III—*caspase-1/11*^−/−^ HFD vs. *caspase-1/11*^−/−^ HFD, IV—WT HFD vs. WT HFD, and V—*caspase-1/11*^−/−^ HFD vs. WT HFD. **(G)** PCoA plot weighted_unifrac and **(H)** PCoA plot unweighted_unifrac, purple: WT HFD, red: *caspase-1/11*^−/−^ HFD. The tests of significance were performed using a two-sided Student's two-sample *t* test, the non-parametric *p*-values were calculated with Bonferroni correction. *p*-values for comparisons of Distance boxplots **(A)**: I vs. II *p* = 0.09, I vs. III *p* = 1, I vs. VI *p* = 1, I vs. V *p* = 0.05, II vs. III *p* = 0.07, II vs. IV *p* = 0.6, II vs. V *p* = 1, III vs. IV *p* = 1, III vs. V *p* = 0.12, and IV vs. V *p* = 0.54; **(B)**: I vs. II *p* = 0.01, I vs. III *p* = 0.39, I vs. VI *p* = 0.89, I vs. V *p* = 0.01, II vs. III *p* = 0.01, II vs. IV *p* = 1, II vs. V *p* = 1, III vs. IV *p* = 0.03, III vs. V *p* = 0.01, and IV vs. V *p* = 1; **(E)**: I vs. II *p* = 0.010, I vs. III *p* = 1, I vs. VI *p* = 1, I vs. V *p* = 0.01, II vs. III *p* = 0.1, II vs. IV *p* = 0.01, II vs. V *p* = 1, III vs. IV *p* = 1, III vs. V *p* = 0.15, and IV vs. V *p* = 0.01; **(F)**: I vs. II *p* = 0.01, I vs. III *p* = 1, I vs. VI *p* = 1, I vs. V *p* = 0.01, II vs. III *p* = 0.01, II vs. IV *p* = 0.01, II vs. V *p* = 1, III vs. IV *p* = 1, III vs. V *p* = 0.01, and IV vs. V *p* = 0.01.

We then compared the groups with each other (β-diversity), providing a measure of distance and dissimilarity among samples. Analyzing the effects of diet on *Caspases 1-11*^−/−^ mice only, our data suggest clustering of microbial communities according to diet (Figures 4A,B), considering weighted or unweighted Unifrac. However, if we focus on the weighted Unifrac, which takes into consideration the relative abundance of the bacterial taxa, limiting the impact of low abundance bacteria, we could better associate it with a percentage of variance found in the PCA plot (Figures 4C,D). If we focus on the effects of the absence of caspases 1 and 11 after a HFD compared to WT mice, we observed a significant difference in the β-diversity between these groups (Figures 4E,F). In this latter case, the clustering is more evident with the unweighted Unifrac, suggesting that the presence or absence of certain bacterial taxa is more important than their relative abundance (Figures 4G,H).

**Figure 5:**
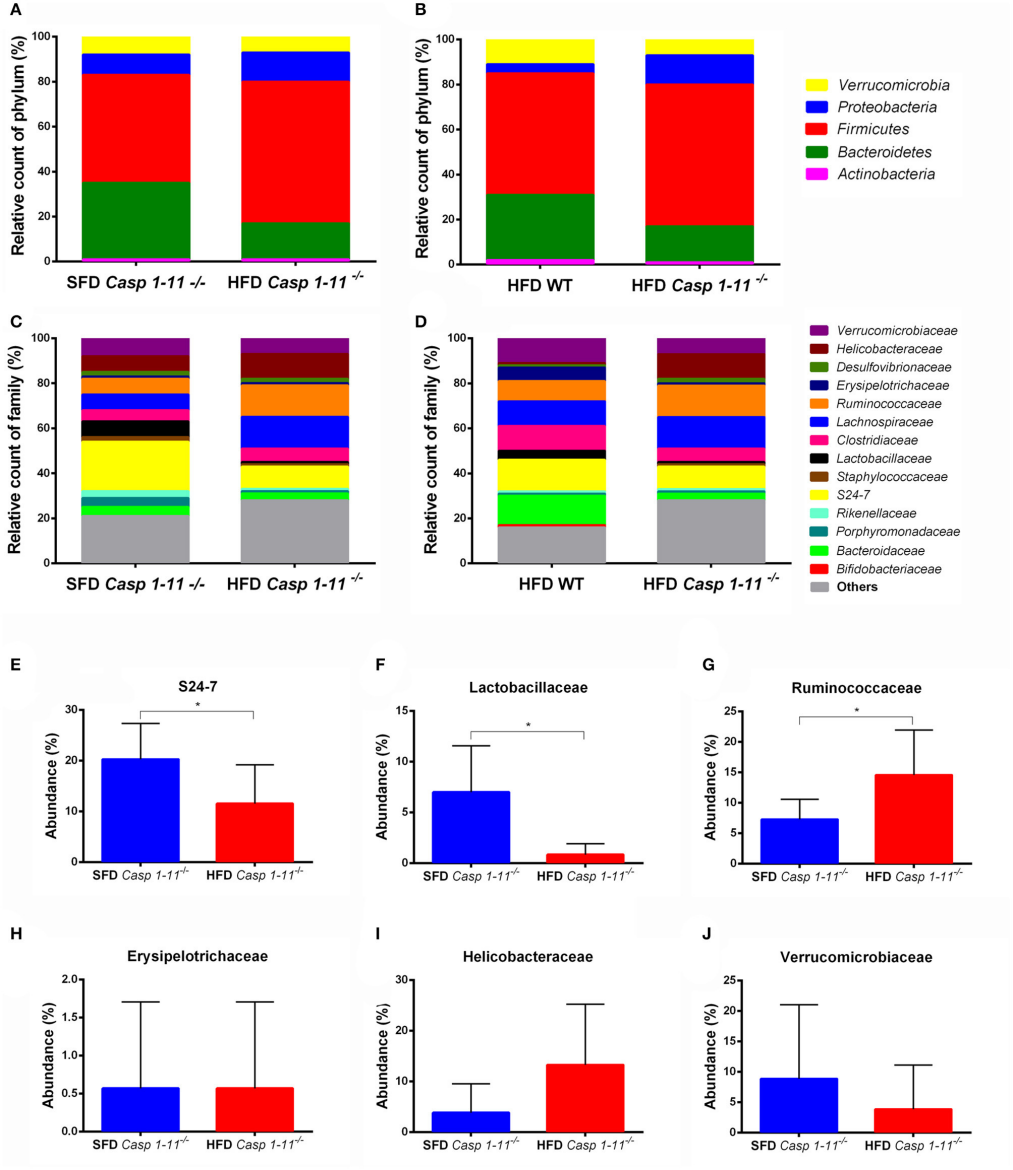
Taxonomic analysis of the gut microbiota. WT and *caspase-1/11*^−/−^ mice were fed a SFD (17% fat) or a HFD (45% fat) for 90 days. Feces from all groups were collected and 16S rRNA gene sequencing of the V4 region was performed on the Illumina HiSeq platform to characterize the distal gut microbiota. Bar charts represent the relative abundance of bacterial **(A,B)** phyla and **(C,D)** families in all mice according to their diet and genotype (*n* = 481 samples). Differences in the abundance of a subset of bacterial families related to NAFLD found in the gut of *Caspases 1-11*^−/−^ mice, according to their diet **(E–J)**. Significance was obtained with a *t* test for each bacterial taxa, the bars represent confidence interval of 95% (*p < 0.05).

Consistent with previous reports (26), our taxonomic analysis identified the *Firmicutes, Bacteroidetes, Proteobacteria, Verrucomicrobia*, and *Actinobacteria* as the most abundant phyla in mice fed with both types of diets (Figures 5A,B). A significant change in bacterial community composition at the phylum level was observed in the *Caspases 1/11*^−/−^ mice fed a HFD compared to *Caspases 1/11*^−/−^ fed a SFD. *Caspases 1/11*^−/−^ mice fed a SFD had highest levels of *Firmicutes* (47.7%), followed by *Bacteroidetes* (33.8%), *Proteobacteria* (9.2%), *Verrucomicrobia* (3.2%), and *Actinobacteria* (1.1%), whereas *Caspases 1/11*^−/−^ mice fed a HFD had increased levels of *Firmicutes* (62.6%), *Proteobacteria* (13.3%), and *Verrucomicrobia* (6.8%), and lower levels of *Bacteroidetes* (16.4%) and *Actinobacteria* (0.5%). We also observed alterations between the WT and *Caspases 1/11*^−/−^ mice fed a HFD, with 54% *Firmicutes*, 29.4% *Bacteroidetes*, 3.8% *Proteobacteria*, 10.9% *Verrucomicrobia*, and 1.5% *Actinobacteria*.

*Caspases 1/11*^−/−^ mice fed a HFD presented a higher *Firmicutes/Bacteroidetes* ratio (F/B), equal to 3.81, compared to WT mice where F/B were 1.84. This was consistent with the highest weight gain observed in *Caspases 1/11*^−/−^ mice fed a HFD (Figure 1) and previous reports (6). The *Caspases 1/11*^−/−^ mice fed a SFD, which also presented initial liver steatosis, showed higher amounts of *Proteobacteria*, a phylum already associated with obesity and NASH (27).

At the family level, we also observed differences when analyzing the effect of the HFD in the *Caspases 1/11*^−/−^ and WT mice (Figures 5C,D). The levels of the *Verrucomicrobiaceae* family after HFD decreased from 10.9% in WT mice to 6.8% in *Caspases 1/11*^−/−^ mice. The *Erysipelotrichaceae* family also decreased in *Caspases 1/11*^−/−^ mice fed a HFD (1%) compared to WT mice under the same diet (6.4%). A similar event was also observed for the *Lactobascillaceae* in the *Caspases 1/11*^−/−^ fed a HFD mice (1.2%) compared to WT mice fed a HFD (4%). In contrast, the *Ruminococcaceae* family increased in *Caspases 1/11*^−/−^ mice fed a HFD (14.4%) compared to WT under the same diet (8.5%). Likewise, *Helicobacteraceae* increased from 1.3% in *Caspases 1/11*^−/−^ mice fed a HFD compared to 10.9% in WT mice fed a HFD.

There was no significant difference in overall β-diversity when we analyzed *Caspases 1/11*^−/−^ mice fed a SFD or HFD. However, some specific families were significantly different in mice fed each diet. We observed a decrease of the *S24-7* and *Lactobascillaceae* (Figures 5E,F) and an increase of the *Ruminococcaceae* families (Figure 5G) in the *Caspases 1/11*^−/−^ mice fed a HFD compared to the same mice fed a SFD. However, there was no significant difference for *Erysipelotrichaceae, Helicobacteraceae*, and *Verrucomicrobiaceae* families in these same conditions (Figures 5H–J).

## Discussion

Inflammation plays a fundamental role in the pathophysiology of obesity, and pro-inflammatory cytokines IL-1β and IL-18 have already been linked to this metabolic disorder. Since caspase 1 is an important regulator for the maturation of these cytokines during inflammasome activation, many studies have started to investigate the role of this protein in the development of obesity, NAFLD, and other metabolic disorders. The precise role of caspase-1 during metabolic inflammation is still unclear and remains a little controversial due to contrasting results obtained with the use of knockout mice (28). Previous work from Wang et al. (13) and Kimura et al. (29) found that caspase-1 knockout mice increased their body weight under HFD compared to WT mice. Dixon et al. (30) demonstrated that subcutaneous and total body adipose tissue of caspase 1 knockout mice was also higher compared to WT mice after HFD. In our work, the bodyweight of *Caspases 1/11*^−/−^ mice fed HFD was also higher than WT, consistent with these studies. However, the results reported by Wang et al. (13) also demonstrated sex-specific differences in the weight gain of caspase 1 knockout mice, with female mice gaining weight less rapidly than their male counterparts. Our result showed that *Caspases 1/11*^−/−^ female mice gained weight more rapidly than Wang et al. study showed, reaching 50 g after only 3 months. This potentiated effect could be due to the absence of two caspases in our model. However, additional studies using single caspase 11 knockout mice are necessary to confirm this.

It has been described that the increase of lipid content in the liver, or hepatic steatosis, can be attributed to excessive intake of alcohol or fat that is higher than the liver is able to metabolize, thereby resulting in fat deposition in microvesicles in hepatocytes (31). Steatosis increases the risk of metabolic disorders linked with obesity such as hypertension, diabetes, dyslipidemia and insulin resistance (32), in addition to increasing the risk of cardiac complications and cardiovascular disease mortality (31). A recent study showed that NLRP3 inflammasome activation is needed for the development of hepatic steatosis, where *Nlrp3*^−/−^ mice were protected against hepatomegaly, liver injuries, and infiltration of activated macrophages in a long-term manner (33). Our results support this, as the group of *Nlrp3*^−/−^ mice under HFD presented reduced levels of steatosis compared to WT mice.

Many patients with NAFLD end up progressing to a state of NASH. NAFLD is the most common cause of chronic liver disease in the United States, reaching up to 100 million individuals, of which 25% progress to NASH (34). Trying to understand the role of the inflammasome in NASH progression, Henao-Mejia et al. (15) fed WT, *Asc*^−/−^, and *Caspase 1*^−/−^ mice a methionine-choline-deficient diet (MCDD). Their results showed that *Asc*^−/−^ and *Caspase 1*^−/−^ mice under MCDD presented an enhanced microvesicular and macrovesicular hepatic steatosis. Kimura et al. (29) and Dixon et al. (30) found that visceral and subcutaneous fat contents were increased in *Caspase 1*^−/−^ mice compared to WT. However, they did not report an increase in the lipid content in the liver (29). Our results showing an increase in the liver weight of *Caspases 1/11*^−/−^ mice fed a HFD, which followed the same pattern as total body weight, support these previous studies. In addition, we also showed that the steatosis levels in these mice are greater compared to WT mice on the same diet. It is also important to note that even under the SFD, *Caspase 1*^−/−^ mice already presented higher steatosis than WT.

NAFLD can be triggered by several distinct causes involving interaction of genetic predisposition and metabolic, inflammatory, and environmental factors (35). Among these factors, dysregulation of gut microbiome has been linked to the development of fatty liver disease. Interactions between the gut microbiome and the host can drive both intestinal homeostasis and disease. However, the mechanisms of this cross-talk remain unclear. In the past decade, the substantial role of the gut microbiota in the progression of obesity and NAFLD has been identified, and the role of altered microbial metabolite production or signaling pathways, such as bile acids or choline metabolism, has also been highlighted (36).

Corroborating with studies of Wang et al. (13) and Kimura et al. (29) that showed the susceptibility of *Caspases 1*^−/−^ mice to gain more weight than WT mice, we further showed here an increase in steatosis levels in the liver and difference in the liver global lipid profile in *Caspases 1/11*^−/−^ and *Nlrp3*^−/−^ mice fed a SFD and a HFD. We identified that the obesity state of *Caspases 1/11*^−/−^ mice resulted in the most altered phenotype, with a complete change in the liver lipid profile, as well as a significant increase in steatosis. This is consistent with the changes in gut microbial diversity, where we also observe a greater proportion of *Firmicutes* and a relatively lower proportion of *Bacteroidetes*. Kimura et al. (29) found that HFD promoted the infiltration of inflammatory macrophages through the CCL2/CCR2 axis, intensifying the development of obesity in *Caspases 1*^−/−^ mice. Here, we demonstrate that this inflammatory context is enriched by alterations in the gut–liver axis, highlighting the major changes in lipid profile and gut microbial composition. Moreover, gut dysbiosis can lead to endotoxemia and inflammation of the gut wall and activation of Kupffer cells and hepatic stellate cells leading to liver injury and inflammation (37). Backhed et al. (8) demonstrated that microbiome transplantation from conventional mice to GF mice produced a 57% increase in total body fat content and a 2.3-fold increase in the content of liver triglycerides in GF mice. Gut colonization triggered an increase of the mRNA levels of two key enzymes for the fatty acid biosynthetic pathway: acetyl-CoA carboxylase (also known as Acc1) and fatty acid synthase (also known as Fas). This may also indicate how gut microbiota modifications could modulate liver lipid content leading to steatosis.

While the F/B ratio can be used as an indicator of obesity, we also report changes in pro-inflammatory and/or putative pathogenic bacterial taxa. *Proteobacteria* are generally associated with endogenous alcohol production and metabolism in conditions of oxidative stress and are substantially increased in steatosis and NASH patients (27). Our results show that the *Caspases 1/11*^−/−^ animals, even under SFD, presented higher levels of *Proteobacteria* in their gut, which were even higher in mice fed a HFD that showed increased weight gain and steatosis.

The role of *Verrucomicrobia* and *Actinobacteria* in obese is very controversial in the literature. In our study, the HFD decreased the levels of *Verrucomicrobia* and *Actinobacteria* in *Caspases 1/11*^−/−^ mice compared to the same mice fed a SFD. However, our results contrast with previous reports, where *Actinobacteria* increased in obese subjects (38, 39). It has also been shown that the abundance of *Verrucomicrobia* was reduced in obese individuals (40), while another study showed that it was increased in with diet-induced obese animals (39). This demonstrates the need for further studies of gut microbiota at the species level to deeper understand its role in obesity.

As mentioned here before, gut–liver axis cross-talk can be highly mediated by inflammatory pathways. Our results also suggest that inflammatory components can be directly associated with changes in bacterial phyla observed in the mice analyzed here. Dysbiosis involving the *Lactobascillaceae* family in the context of steatosis was already described in cases where liver diseases were associated with alcohol (41). In the present study, we observed that *Caspases 1/11*^−/−^ mice fed a HFD presented the most severe liver steatosis, the highest weight gain, and the lowest amount of *Lactobascillaceae* in the gut microbiota. Our findings corroborate with Wang and colleagues (42) that demonstrated that abundance and prevalence of the *Lactobascillaceae* family were reduced in the gut microbiota of NAFLD patients. *Caspases 1/11*^−/−^ mice fed a HFD also presented an increase for S24-7, a family of bacteria within the order Bacteroidales, already correlated with obesity cases (43).

It is interesting to note that the absence of caspases 1 and 11 was sufficient to significantly change not only the liver steatosis levels but also the composition of gut microbiota, not regarding the role of the diets. Indeed, the *Helicobacteraceae* and *Ruminococcaceae* families were more abundant in *Caspases 1/11*^−/−^ mice fed a SFD, and this effect was intensified when these mice were fed a HFD. The increased abundance of the *Ruminococcaceae* family has also been reported in a study with obese children (44) and women (43).

Our study suggests that the effector caspases 1 and 11 have a function on the liver–gut axis cross-talk, playing a substantial role in the pathogenesis of obesity. Taken together, our data suggest an important role for caspases 1/11 in the global lipid composition of the liver and in the modulation of the gut microbial community composition. Our results further suggest that HFD-induced obesity in addition to the absence of caspase 1/11 may regulate both liver global lipid metabolism and gut microbial diversity, strongly favoring the establishment of the NAFLD and obesity. Moreover, our data support the sense that manipulating gut microbiota could have a therapeutic potential to decrease the incidence and prevalence of fatty liver diseases and obesity.

## Data Availability Statement

The raw data supporting the conclusions of this manuscript will be made available by the authors, without undue reservation, to any qualified researcher.

## Ethics Statement

The animal study was reviewed and approved by CEUA (Comitê de Ética em Uso de Animais) da Universidade de Brasilia (UnBDoc n 52306/2014).

## Author Contributions

KM and LS: conceptualization. KM, ME, and CM: supervision. LS, DR, RC, RA, AM, and FS: formal analysis. DR: writing and editing. KM and CM: revision. KM: writing and editing and funding acquisition. All authors read and approved the final manuscript.

### Conflict of Interest

The authors declare that the research was conducted in the absence of any commercial or financial relationships that could be construed as a potential conflict of interest.
