# Supplementary figures and images for: Absence of the Caspases 1/11 Modulates Liver Global Lipid Profile and Gut Microbiota in High-Fat-Diet-Induced Obese Mice
Source: Front Immunol. 2020 Jan 9;10:2926. doi: 10.3389/fimmu.2019.02926 (PMC6962112; doi:10.3389/fimmu.2019.02926)

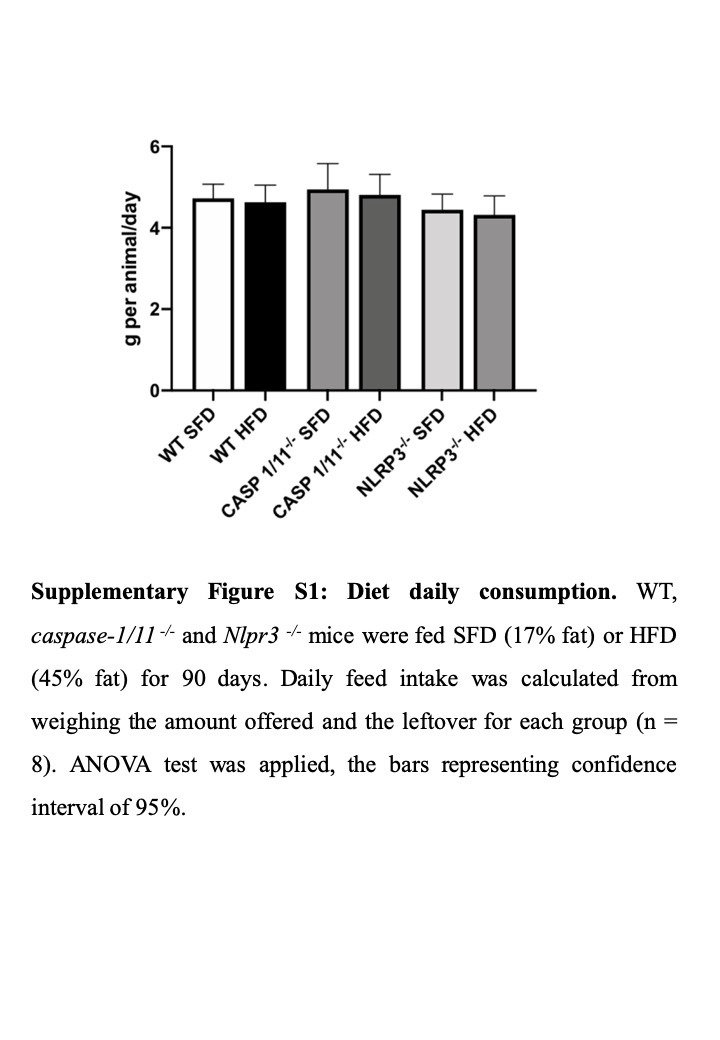

Supplement: Supplementary file 1 [file Image_1.jpg]

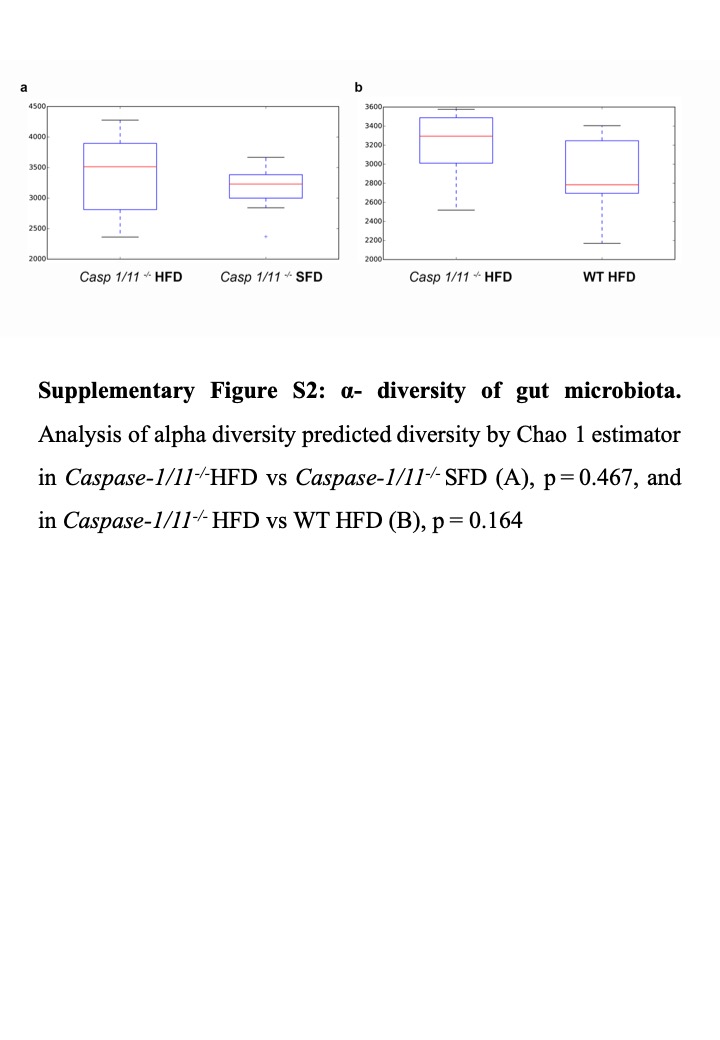

Supplement: Supplementary file 2 [file Image_2.jpg]
